# Biochar regulates putative keystone microbial taxa to drive phosphorus cycling and increase availability in urban greenspace soils

**DOI:** 10.3389/fmicb.2026.1786258

**Published:** 2026-03-13

**Authors:** Kai Pan, Zhenying Zhang, Lingwei Feng, Xiaogang Wu, Xiuyun Yang, Xinping He, Yiqian Xiao, Danning Yang, Chengjiao Duan, Qiang Wang

**Affiliations:** 1 College of Forestry, Shanxi Agricultural University, Taigu, China; 2 China Highway Engineering Consultants Corporation, Beijing, China; 3 College of Resources and Environment, Shanxi Agricultural University, Taigu, China; 4 State Key Laboratory of Efficient Utilization of Arid and Semi-Arid Arable Land in Northern China, Institute of Agricultural Resources and Regional Planning, Chinese Academy of Agricultural Sciences, Beijing, China

**Keywords:** biochar, greenspace soil, metagenomics, phosphorus cycling, putative keystone microbial taxa

## Abstract

The quality of soil in urban green spaces often deteriorates due to poor design practices, insufficient maintenance, and environmental pressures associated with urbanization. Although biochar, as an effective soil additive, can significantly improve the soil quality in greenspace, it significantly influences the phosphorus (P) cycling processes through functional regulation of microbial community; however, further analysis is essential to validate this mechanism. Therefore, this study reported pot experiments using *Euonymus kiautschovicus*, a typical urban greenspace plant, followed by metagenomic analysis for investigating microbial-driven P cycle mechanisms. Four treatment groups were established according to the dosage of biochar, including 0% (CK), 4% (BC4), 8% (BC8), and 12% (BC12). Biochar application significantly increased soil available P (AP) and total P (TP) content, with BC12 demonstrating maximum AP and TP content of 21.79 mg kg^−1^ and 0.62 g kg^−1^, respectively. On the one hand, biochar serves as a direct source of P. On the other hand, it enhances AP by regulating P-cycling functional microorganisms. Random forest model identified *phnP*, *phoA*, *relA*, *ppnK*, *pstA*, *phnD*, and *pstS* as the putative keystone genes regulating soil P cycling. Microbial co-occurrence network analysis and partial least squares path modeling (PLS-PM) demonstrated that the biochar application improved soil AP by regulating putative keystone microbial taxa (Modules 1 and 2) involved in P cycling. This study elucidates the microbial mechanisms underlying biochar-mediated P cycling in greenspace soils, providing a scientific basis for biochar application for improved soil quality in urban greenspace.

## Graphical abstract

**Figure:**
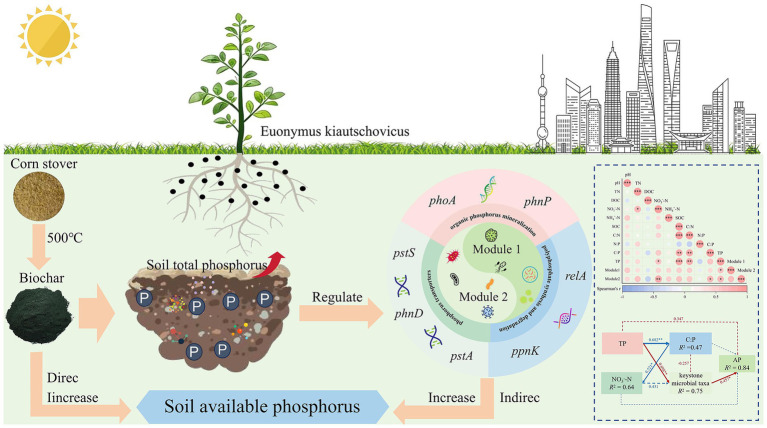

## 1 Introduction

Urban greenspace refers to urban wetlands, urban forests, and urban lawns (Guo et al., 2024). Soils within urban greenspaces contribute substantially to terrestrial ecosystems, urban greenspace soil plays a key role in mitigating climate change and maintaining biodiversity (O'Riordan et al., 2021). However, rapid urbanization decreased the urban greenspace area and led to the deterioration of soil quality (Masson et al., 2020). Furthermore, the intrinsically low nutrient status of urban soils, coupled with intensifying environmental stressors, has substantially weakened the ecological functions and services of urban greenspace (Fekete et al., 2024), leaving them highly vulnerable to degradation. Biochar is a carbon-rich porous material derived from biomass under high-temperature and oxygen-limited conditions, with extensive application as a soil amendment for improved soil quality. Biochar has emerged as a promising strategy for promoting agricultural sustainability (Kalakodio et al., 2018; Li et al., 2023). It is characterized by abundant nutrients, porous and irregular surface structure, high specific surface area, and strong adsorption capacity, enabling efficient retention of both nutrients and pollutants (Zhao et al., 2020). Therefore, it contributes to soil fertility by restoring essential nutrients and strengthening soil structure, improving its capacity to support sustainable plant growth (Wang et al., 2025; Wang et al., 2019; Zhang et al., 2019). Furthermore, biochar, as a porous carbon (C), provides a favorable habitat to microorganisms essential for their growth (Gul and Whalen, 2016; Liao et al., 2022), and contributes significantly to C sequestration and stability of the soil ecosystem. These properties render biochar a highly suitable soil amendment for urban greenspace (Niu et al., 2024).

The positive influence of incorporating carbon-based materials on soil phosphorus (P) availability has been previously reported (Damon et al., 2014), with straw or organic amendments, including compost and biochar, demonstrating significantly increased soil P availability (Pu et al., 2023). Biochar is inherently alkaline and increases the soil pH after application, creating a more favorable environment for P-solubilizing microorganisms capable of influencing the soil P availability (Oburger et al., 2011). Moreover, soil P availability is controlled by physicochemical processes, including adsorption–desorption dynamics and pH-dependent precipitation-dissolution. Furthermore, organic P mineralization significantly regulates P availability in soil (Gosling and Shepherd, 2005; Chang et al., 2025). Soil microorganisms perform central role in P cycling through a variety of functions. Microbial P transformation can be categorized into five theoretically defined processes: organic P mineralization (OPM), inorganic P solubilization (IPS), polyphosphate synthesis and degradation (PSD), P cycling regulation (REG), and P transporters (TRA) (Santos-Beneit, 2015). The microorganisms harboring OPM-related genes can effectively mineralize organic P compounds *via* secretion of various phosphatases, such as alkaline phosphatase encoded by *phoA* and *phoD*, phytase encoded by *phnG*, and *phnH* (Xie et al., 2025). Microbes involved in the IPS process typically carry functional genes, such as *gcd*, *ppa*, and *ppx*, enabling the regulation of insoluble phosphates through the secretion of organic acids, such as citric, gluconic, oxalic, and succinic acid, along with hydrogen ions (Peng et al., 2025; Zhang et al., 2023). Furthermore, the genes encoding phosphate transporters (*pst*), phosphonate transporters (*phnC*, *phnD*, *phnE*), and phosphoglycerate transporters (*pgt*) demonstrated significant participation in P uptake and transport (Huang et al., 2024). However, the mechanisms through which P-cycling microorganisms regulate P transformation processes following biochar amendment in greenspace soils remain poorly understood.

This study aims to elucidate the microbial-driven processes through which biochar regulates soil P cycling in greenspace soils using the combined greenfield plant pot experiments with metagenomic techniques. We hypothesize that the biochar amendment would influence the soil physicochemical properties affecting the microbial community involved in P cycling, increasing soil P availability. This research provides a deeper understanding of the relationships of biochar with P-cycling microorganisms.

## 2 Materials and methods

### 2.1 Study site

The experiment was conducted at the Shanxi Agricultural University Nursery in Taigu County, Jinzhong City, Shanxi Province, China (112°28′E; 37°12’N; Altitude: 799 m), located in an arid to semi-arid region, with a warm temperate continental climate. The site is characterized by an annual average temperature of 9.8 °C, an effective accumulated temperature ≥ 10 °C: 3675 °C, an annual sunshine duration of 2738.5 h, a frost-free period of 176 days, an annual average precipitation of 450–500 mm, and an annual average evaporation of 1765.9 mm.

### 2.2 Materials

A typical, nutrient-deficient, and unpolluted urban greenspace soil samples were collected around Shanxi Agricultural University. The following are the basic physicochemical properties of soil: pH = 8.40; soil organic matter (SOM) of 9.63 g kg^−1^; total nitrogen (TN) of 0.25 g kg^−1^; total phosphorus (TP) of 0.40 g kg^−1^; and AP of 7.29 mg kg^−1^. Corn stover biochar, derived through the pyrolysis at 500 °C under oxygen-limited conditions, was used as a soil amendment. The following are the characteristics of the employed biochar: particle size of ≤ 2 mm; pH 9.92, organic C content of 511 g kg^−1^, TN of 8.51 g kg^−1^, TP of 2.34 g kg^−1^, and AP of 11.13 mg kg^−1^. Annual seedlings of *Euonymus kiautschovicus*, a representative urban greenspace species with an average height of 30 ± 5 cm, were used for the pot experiment. All seedlings were carefully selected based on vigorous growth and confirmed to be free of pests/diseases.

### 2.3 Experiment design

The biochar application rates were determined in accordance with previous literature Fan et al. (2020). Four treatment groups were established based on varying volume ratios: CK (0% biochar), BC4 (4% biochar), BC8 (8% biochar), and BC12 (12% biochar). Each treatment group was replicated four times.

PVC tubes (45 cm diameter × 100 cm height) filled with 150 kg of uniformly sieved soil (<2 mm) were used as pot containers, with each pot containing soil to a depth of 85 cm. Biochar was incorporated into the top 30 cm of soil and thoroughly mixed, and soil moisture was maintained at 60% field capacity for three months, followed by planting of *Euonymus kiautschovicus* seedlings on July 22, 2023. All the potted plants were positioned outdoors in an open area with full sunlight exposure.

### 2.4 Soil sampling

Soil samples were collected in September 2024 after one year of the pot experiment. Surface samples (0–30 cm depth) were randomly collected from each pot, with four replicates per group. The collected samples were divided into two parts, with one portion sieved (2 mm) and stored at −80 °C for metagenomic analysis. The remaining soil was air-dried in shade and sieved through 1 mm and 0.149 mm meshes for the determination of physicochemical properties.

### 2.5 Samples measurement

Soil pH was measured using a pH meter (Shanghai Leici PHS-3C) in a water-soil suspension (2.5:1). Soil organic C (SOC) was determined using the K_2_Cr_2_O_7_ oxidation-FeSO_4_ titration method, while the quantification of SOM was carried out through the multiplication of SOC by a factor of 1.724, as per the standard conversion protocols (Wang et al., 2025). TN content in soil was determined using a continuous flow analyzer (AA3, SEAL, Germany). The ammonium (NH_4_^+^-N) and nitrate (NO_3_^−^-N) concentrations were determined by extracting soil samples with 1 mol L^−1^ KCl, followed by analysis of the extracts using the same continuous flow analyzer (Zhang et al., 2024). Soil AP was extracted with NaHCO_3_ (pH = 8.5) and quantified using the molybdenum–antimony colorimetric method (Duan et al., 2022). TP content was measured using HClO_4_-H_2_SO_4_ digestion followed by analysis through the same colorimetric method (Qi et al., 2025). Soil dissolved carbon (DOC) was extracted by 0.5 mol/L K_2_SO_4_ and determined through a total organic carbon analyzer (TOC-VCPH, Shimadzu, Japan) (Jones and Willett, 2006).

Biochar samples were analyzed according to the method reported by Wang et al. (2023). TN was determined using Raney’s alloy macro method, organic carbon was measured by the potassium dichromate volumetric method, and TP was determined by H_2_SO_4_-H_2_O_2_ digestion using ultraviolet spectrophotometer analysis. The pH was determined 24 h after the addition of biochar to 20 mL of CO_2_-free deionized water using a Mettler Toledo FE20 pH meter. The AP content of biochar was determined spectrophotometrically using molybdovanadophosphoric acid.

### 2.6 DNA extraction and sequencing

DNA extraction was performed using the Mag-Bind® Soil DNA Kit (Omega Bio-tek, USA) according to the manufacturer’s protocol. DNA integrity was verified using 1% agarose gel electrophoresis, and qualified DNA samples were sent to Shanghai Majorbio Bio-pharm Technology Co., Ltd. (http://www.major.bio.com) for metagenomic sequencing using the Illumina NovaSeq platform (Illumina Inc., USA).

### 2.7 Analysis of sequence data

The processing of raw sequencing data was carried out using the Majorbio Cloud Platform (Majorbio, Shanghai, China), while Data quality control was performed using fastp software (version 0.20.0), which trimmed adapter sequences from the 3′ and 5′ ends of reads, neglecting the reads shorter than 50 bp or with an average base quality score < 20. The optimized sequences were assembled using MEGAHIT (version 1.1.2), and open reading frames (ORFs) within the assembled contigs were predicted using Prodigal. Genes with ≥ 100 bp were selected for translation into the respective amino acid sequences. Finally, the predicted gene sequences from all samples were clustered using CD-HIT (version 4.6.1) to construct a non-redundant gene set, comprising 4,310,984 unique genes. The non-redundant gene sets were aligned against the KEGG gene database using BLASTP (Version 2.2.28), and functional annotation was subsequently performed with the KEGG Orthology-Based Annotation System (KOBAS 2.0). Gene function analysis was performed by calculating the TPM value for each gene to normalize their abundance. To facilitate further analysis, functional annotation of the sequences obtained from each sample was performed using the Kyoto Encyclopedia of Genes and Genomes (KEGG) database (http://www.genome.jp/kegg). P-cycling gene taxonomy were performed according to the protocol described by Wang et al. (2024). A total of 49 P-cycling genes were screened and categorized into five functional groups, including OPM, IPS, REG, TRA, and PSD, followed by the analysis of the total abundances of the corresponding KO genes.

### 2.8 Statistical analysis

Data statistics and processing were performed using Excel 2021, and one-way analysis of variance (ANOVA) was performed using IBM SPSS 27.0 software (IBM Inc., USA). Significant differences between treatment groups were determined using Duncan’s test at a significance level of *p* < 0.05. The bioinformatic analysis of sequence data was carried out using the Majorbio Cloud platform (https://cloud.majorbio.com), while the determination of the *α*-diversity indices of soil microbial communities was performed through Mothur software. Non-metric multidimensional scaling (NMDS) based on Bray-Curtis distance was used to examine the *β*-diversity characteristics of P-cycling functional genes and microorganisms. Random forest analysis was performed using the “randomforest” and “rfPermute” packages of R 4.4.2, aiming to identify the putative keystone P-cycling genes. Co-occurrence networks were constructed based on Spearman’s correlations using the “psych” and “igraph” packages, while network visualization and Module detection were performed using Gephi 0.10.1. The rest of the figures were generated using Origin 2024 (Origin Lab Inc., MA, USA).

## 3 Results

### 3.1 Effects of biochar on soil basic properties

Biochar application increased the soil pH, SOC, TN, TP, C:N, C:P, NO_3_^−^-N, and AP contents in urban greenspace soil (Table 1). The BC4 treatment significantly increased soil SOC, TN, TP, C:P, NO_3_^−^-N, and AP contents upon comparison with CK. Furthermore, the BC8 treatment significantly increased soil SOC, TN, TP, C:N, C:P, NO_3_^−^-N, and AP contents, while the BC12 treatment further elevated soil pH in addition to these properties (Table 1). With increasing application rate of biochar, the SOC, TN, TP, C:N, C:P, NO_3_^−^-N, and AP contents increased gradually, achieving a maximum value for BC12 treatment with respective values of 11.62 g·kg^−1^, 0.58 g·kg^−1^, 0.62 g·kg^−1^, 19.98, 18.62, 7.40 mg·kg^−1^, and 21.79 mg·kg^−1^ (Table 1).

**Table 1:** Relative abundance of putative keystone functional genes for P cycling across.

Processes	KEGG name	Relative abundance of putative keystone P cycling genes (%)			
		CK	BC4	BC8	BC12
OPM	*phnP*	1.16 ± 0.13b	1.17 ± 0.10b	1.21 ± 0.13b	1.57 ± 0.19a
	*phoA*	2.95 ± 0.29b	2.87 ± 0.25b	3.18 ± 0.10b	3.87 ± 0.41a
PSD	*relA*	0.94 ± 0.02b	0.84 ± 0.06b	0.95 ± 0.07b	1.33 ± 0.21a
	*ppnK*	3.95 ± 0.40b	4.28 ± 0.46b	4.52 ± 0.14ab	5.10 ± 0.69a
TRA	*pstA*	4.37 ± 0.31b	4.50 ± 0.26b	4.80 ± 0.17ab	5.28 ± 0.63a
	*phnD*	1.38 ± 0.21b	1.39 ± 0.22b	1.41 ± 0.10b	1.78 ± 0.19a
	*pstS*	6.70 ± 0.54b	6.75 ± 0.54b	7.26 ± 0.59ab	8.27 ± 1.09a

CK, control; BC4, 4% biochar; BC8, 8% biochar; BC12, 12% biochar; OPM, organic phosphorus mineralization; PSD, polyphosphate synthesis and degradation; TRA, phosphorus transporters. Lowercase letters indicate significant differences among treatments based on Duncan’s post hoc test at the level of *P* < 0.05. Values represent the mean ± standard deviation (*n* = 4).

### 3.2 Effects of biochar on P-cycle microbial communities and functional genes

**Figure 1:**
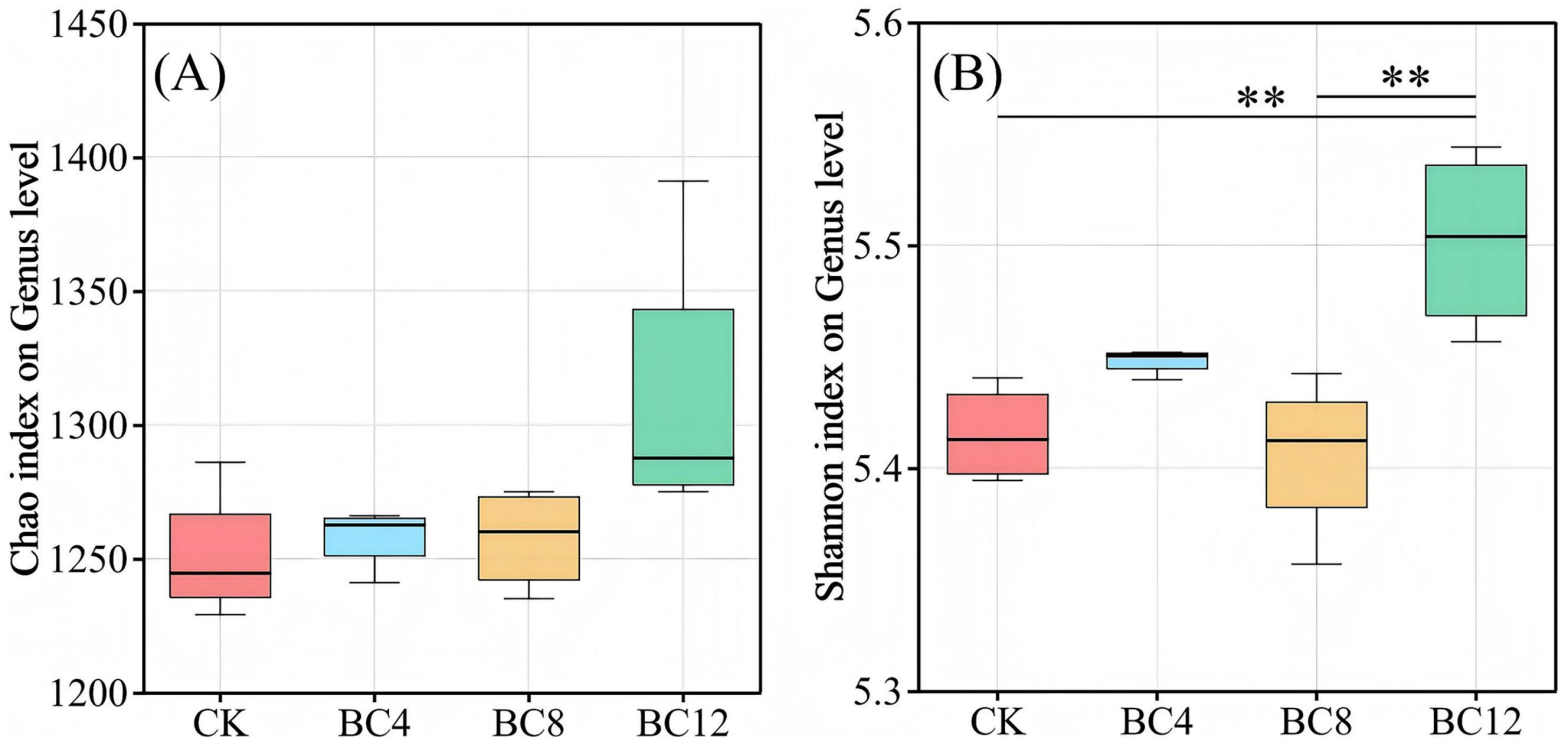
Differences in *α*-diversity of soil microbes across different treatments. Note: CK, control; BC4, 4% biochar; BC8, 8% biochar; BC12, 12% biochar. **p < 0.01. Values represent the mean ± standard deviation (*n* = 4).

The variations in the *α*-diversity of soil P-cycling microbes among the treatment groups are shown in Figure 1. At the genus level, the BC12 treatment demonstrated a significantly higher species Shannon index compared to CK and BC8 (Figure 1). NMDS analysis indicated that the biochar application altered the P-cycling functional microbial community structure (stress = 0.091, Supplementary Figure S1A) and associated gene structure in urban greenspace soils to a certain extent (stress = 0.026, Supplementary Figure S1B).

**Figure 2:**
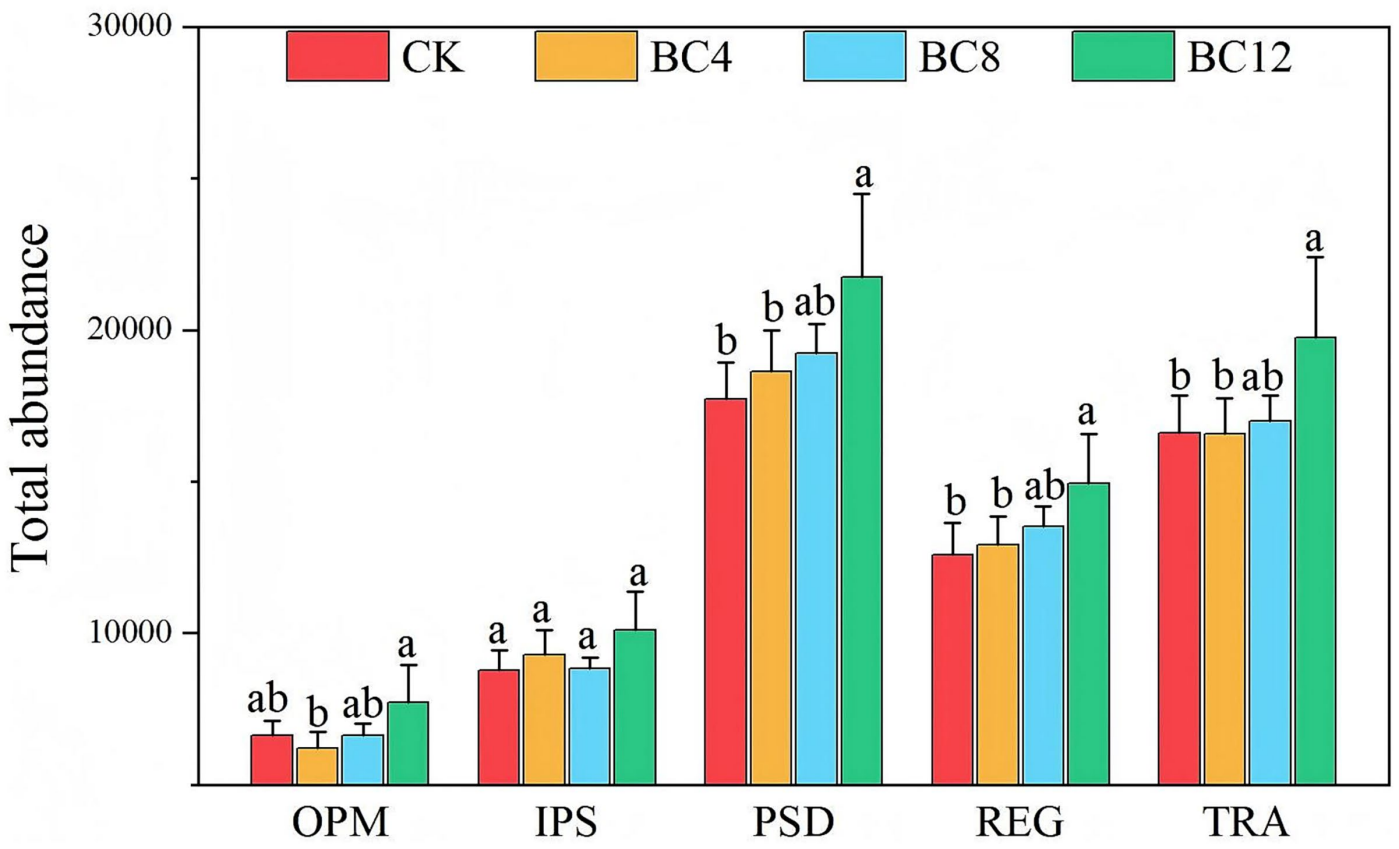
Effects of biochar on the total TPM abundance of P-cycling functional genes. Note: CK, control; BC4, 4% biochar; BC8, 8% biochar; BC12, 12% biochar. OPM, organic phosphorus mineralization; IPS, inorganic phosphorus solubilization; PSD, polyphosphate synthesis and degradation; REG, phosphorus cycling regulation; TRA, phosphorus transporters. Lowercase letters indicate significant differences among treatments based on Duncan’s *post hoc* test at the level of *p* < 0.05. Values represent the mean ± standard deviation (*n* = 4).

As shown in Figure 2, the abundance of genes involved in P cycling processes, OPM, PSD, REG, and TRA varied significantly across the different treatments (*p* < 0.05). Furthermore, the BC12 treatment significantly increased the total abundance of genes associated with the PSD, REG, OPM, and TRA processes (*p* < 0.05) compared with the CK and BC4 groups. However, no statistically significant difference was observed in the total abundance of genes associated with the IPS process across all treatment groups.

### 3.3 Keystone genes involved in soil P-cycling

**Figure 3:**
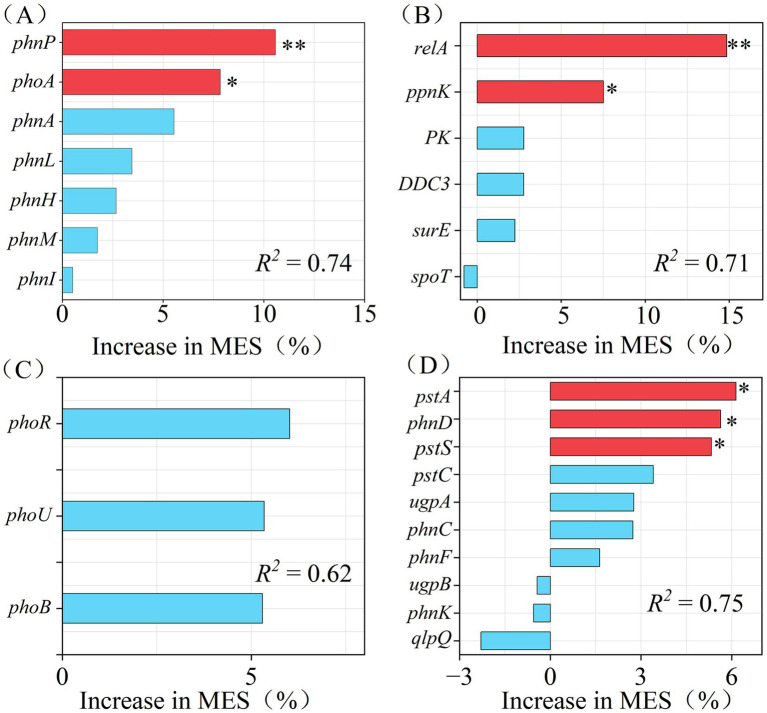
Putative keystone P-cycling functional genes identified by random forest model (*n* = 16). *: *p* < 0.05; **p < 0.01. **(A)** Putative keystone genes in the OPM process; **(B)** Putative keystone genes in the PSD process; **(C)** Putative keystone genes in the REG process; **(D)** Putative keystone genes in the TRA process. The number of decision trees was set to 500, the validation method was ten-fold cross-validation, and the number of permutation tests was 1,000. Note: OPM, organic phosphorus mineralization; PSD, polyphosphate synthesis and degradation; REG, phosphorus cycling regulation; TRA, phosphorus transporters.

Random forest model analysis revealed that the seven functional genes from the OPM process collectively explained 74% of the total variations in AP, with *phnP* identified as the strongest predictor (*p* < 0.01), followed by *phoA* (*p* < 0.05, Figure 3A). Furthermore, six PSD genes demonstrated 71% of the total variations in AP, with *relA* being the optimal predictor (*p* < 0.01), followed by *ppnK* (*p* < 0.05, Figure 3B). Three REG functional genes accounted for 62% of the total variation in AP, although none was identified as a significant predictor (Figure 3C). Finally, a total of ten functional genes in TRA collectively explained 75% variations in AP, with *pstA*, *phnD*, and *pstS* being the optimal predictors (*p* < 0.05, Figure 3D). Therefore, seven functional genes (*phnP*, *phoA*, *relA*, *ppnK*, *pstA*, *phnD*, and *pstS*) across OPM, PSD, TRA processes were identified as the putative keystone P-cycling genes (Figure 3).

Biochar demonstrated a significant effect on the relative abundance of putative keystone P-cycling genes (Table 1), with BC12 treatment showing significantly increased relative abundance of *phnP* and *phoA* genes associated with the OPM process (*p* < 0.05, Table 1), rising from their lowest values of 1.16 and 2.95% to 1.57 and 3.87%, respectively. Furthermore, BC12 significantly increased the relative abundance of PSD-related *relA* and *ppnK* genes, as compared to CK and BC4 (*p* < 0.05, Table 1), demonstrating an increase from 0.94 and 3.95% in CK to 1.33 and 5.1% in BC12, respectively. BC12 significantly increased the relative abundance of *pstA*, *phnD*, and *pstS* genes related to the TRA process compared to CK and BC4 (*p* < 0.05, Table 1), showing the lowest value of 4.37, 1.38, and 6.70% in the CK treatment, which increased to the 5.28, 1.78, and 8.27% in the BC12 treatment. The relative abundance of the seven putative keystone functional genes increased with increasing dosage of biochar application, achieving a maximum value in BC12-treated groups.

### 3.4 Soil P-cycle keystone microbial taxa

**Figure 4:**
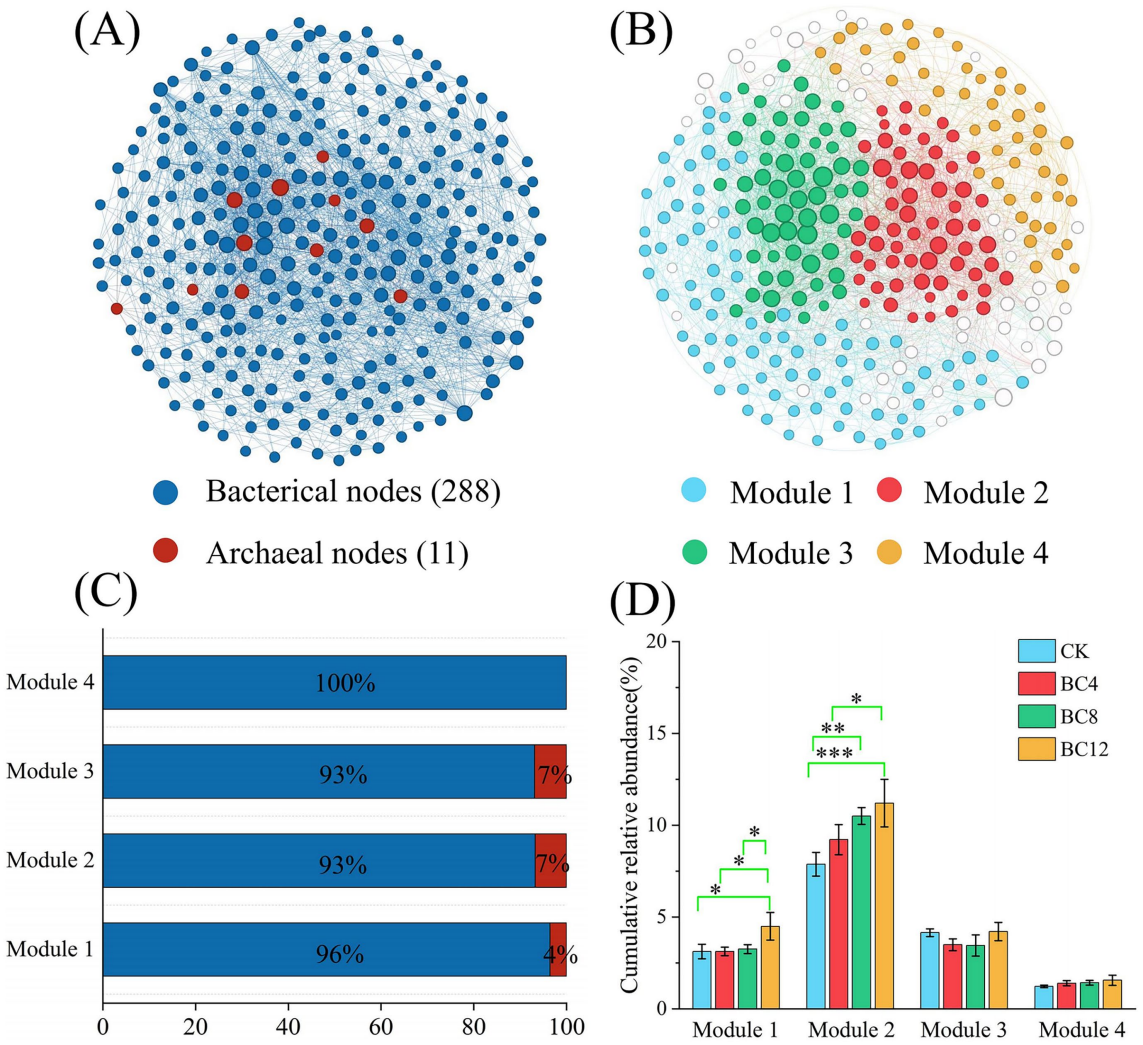
Co-occurrence network of species carrying putative keystone genes in P cycle. **(A)** Co-occurrence network constructed including bacterial and archaeal genera, microbial kingdoms in the network indicated by colors of node (*n* = 16); **(B)** Microbial taxa constructed based on co-occurrence network (Module 1–4), Modules in the network indicated by colors of node (*n* = 16); **(C)** Microbial composition in Module 1–4; **(D)** Cumulative relative abundance of Modules in different samples (*n* = 4). Asterisks indicate statistically significant differences (*p < 0.05; **p < 0.01; ***p < 0.001).

Co-occurrence network module analysis was conducted in accordance with the microbial taxa harboring key P-cycling genes to identify key microbial taxa associated with P cycling (Figure 4). The network was resolved into four major Modules (1–4), accounting for 28.76, 19.73, 19.73, and 16.39% of all nodes, respectively (Figure 4B). Modules 1, 2, and 3 were predominantly comprised of bacterial species (96, 93, and 93%, respectively) with a small archaeal fraction, while Module 4 was solely based on bacterial composition (Figure 4C). In comparison to CK, BC4 and BC8, cumulative relative abundances of Module 1 were significantly higher (*p* < 0.05) in BC12 groups. In Module 2, the cumulative relative abundance was significantly higher under BC8 (*p* < 0.01) and BC12 (*p* < 0.001) compared to CK. Biochar demonstrated no significant effect on the cumulative relative abundance of Modules 3 and 4. Furthermore, Modules 1 and 2 were identified as the most influential, primarily due to the highest number of nodes (Figure 4B) and cumulative abundance (Figure 4D), identifying them as key microbial taxa in the P cycle.

Supplementary Table S2 displays the microbial composition associated with Modules 1 and 2, with Module 1 comprising 82 bacterial genera, showing the predominant genera of *Lysobacter*, *Polaromonas*, *Hyphomicrobium*, *Pseudorhodoplanes*, and *Steroidobacter*. Module 2 comprised 55 bacterial genera, with predominant bacterial genera of *Rokubacteria*, *Sphingomonas*, *Nitrospira*, *Candidatus_Gaiellasilicea*, and *Anaerolinea*.

### 3.5 Effects of soil properties and key microbial taxa on AP

**Figure 5:**
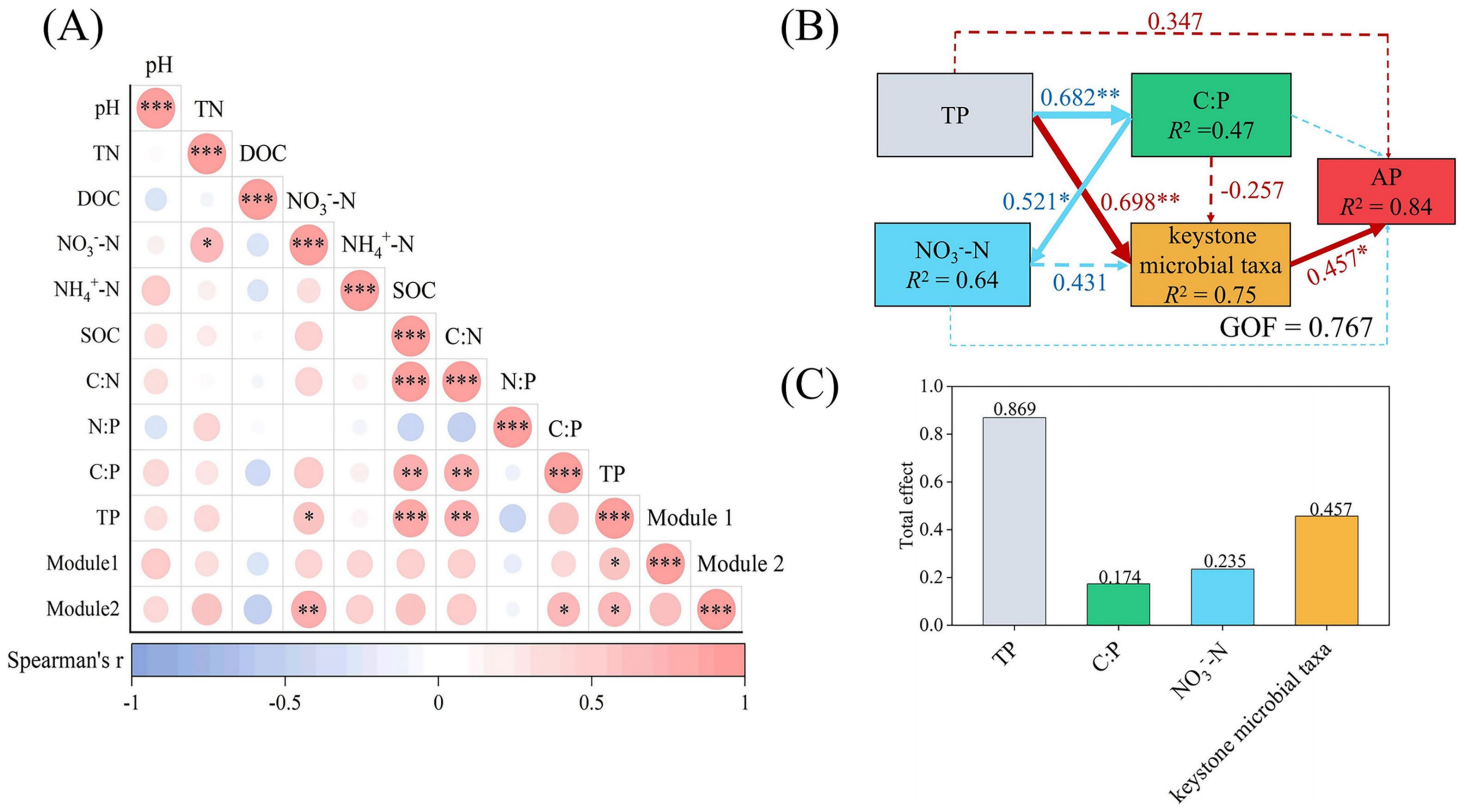
**(A)** Spearman correlations among soil properties and the relative abundances of putative keystone microbial taxa responsible for P cycling in soil (*n* = 16). Significance was adjusted using the Benjamini-Hochberg method. **(B)** Partial least squares path modelling (PLS-PM) disentangling major pathways of the effects of soil properties and putative keystone microbial taxa on AP (*n* = 16). Red and blue arrows indicate positive and negative flows of causality. Solid lines denote significant effects, while dashed lines indicate non-significant effects. Paths with coefficients of absolute values less than 0.2 were omitted. *R*^2^ indicates the dependent variable variance explained by the model. Numbers on the arrow indicate significant standardized path coefficients (*p < 0.05; **p < 0.01); **(C)** as well as the total effects of each variable on AP. Note: SOC, soil organic carbon; TN, total nitrogen; TP, total phosphorus; C:N, SOC/TN; C:P, SOC/TP; N:P, TN/TP; DOC, dissolved carbon; NO_3_^−^-N, nitrate nitrogen; NH_4_^+^-N, ammonium nitrogen; AP, available phosphorus; Soil putative keystone microbial taxa include Modules 1 and 2.

Spearman correlation analysis was performed to evaluate the relationships between microbial taxa (Modules 1 and 2) and soil properties (Figure 5A). Module 1 demonstrated significant correlation with TP (*p* < 0.05), while the relative abundance of Module 2 was determined to be correlated with TP (*p* < 0.05), C:P (*p* < 0.05), and NO_3_^−^-N (*p* < 0.01) (Figure 5A), identifying as the key factors influencing the microbial taxa associated with soil P cycling (Figure 5A).

Based on the Spearman correlation analysis (Figure 5A), PLS-PM was subsequently used to examine the cascading relationships among TP, C:P, NO_3_^−^-N, putative keystone microbial taxa, and AP (GOF = 0.767, Figures 5B,C). The results demonstrated that the TP exerted the highest effect on AP, with a value of 0.869, primarily modulating soil AP content through significant effects on key microbial taxa (Modules 1 and 2) (Figures 5B,C).

## 4 Discussion

### 4.1 Effect of biochar on soil AP

In this study, biochar application significantly increased soil TP and AP content, consistent with previous findings (Yu et al., 2023), indicating the direct input of exogenous P from biochar. The biochar displayed a high inherent P content (TP, 2.34 g kg^−1^; AP, 11.13 mg kg^−1^); its addition directly enhanced the soil TP and AP levels. Furthermore, biochar is abundant in a variety of diverse P forms, including inorganic phosphates, polyphosphates, and organically bound P (Schneider and Haderlein, 2016), which are gradually released through dissolution and desorption processes upon incorporation into the soil, significantly enhancing the soil P pool.

Moreover, by changing soil pH and its physicochemical properties, the soil pH after BC12 treatment was significantly increased, which can be attributed to the strong alkalinity of biochar. Generally, soil pH increased with increasing biochar application (Gerdelidani and Hosseini, 2017). An elevation in soil pH can influence the binding of phosphate and metal ions, leading to a reduction of P fixation in the soil and increasing the soil soluble P content (Chang et al., 2025).

Biochar contains inherent C, enriched nutrients, low density, and a porous structure. It provides a favorable habitat and abundant adsorption sites for P-cycling microorganisms after application to soil, establishing a niche favorable to their growth and proliferation (Shaaban et al., 2018; Wang and Wang, 2019), improving AP content to some extent. Owing to its high specific surface area, abundant micropores, and surface functional groups, biochar indicates strong adsorption capacity for inorganic ions such as phosphates, which have the potential to influence soil AP (Mahmoud et al., 2020). Furthermore, BC12 significantly increased SOC content due to the inherent properties of biochar (such as high organic C content, which serves as a direct C input to soil) (Bekchanova et al., 2024). Modifications to these factors either directly or indirectly influenced the activity of P-cycling soil microorganisms, consequently regulating the AP levels in the soil. Therefore, the direct and predominant mechanism by which biochar amendment enhances soil P lies in its inherent P input and the corresponding pH-mediated physicochemical processes, while the secondary mechanism is represented by modulating microbial communities through altered soil properties.

The soil P content was considerably increased as the biochar dosage increased. For example, BC12 soil indicated a higher P content compared to BC4 and BC8 (Table S1). As a P source input, the initial P content of biochar feedstock directly affects the increase in soil P content (Matin et al., 2020). At lower biochar dosages (such as BC4 and BC8), the TP carried by biochar was limited, and differences in P input among rates were likely masked by background soil P pools, resulting in similar levels of P enhancement. In contrast, BC12 treatment significantly increased the exogenous P input by cumulative effects, generating significant variations in the content of P (Glaser and Lehr, 2019).

### 4.2 Effect of biochar on P-cycling functional genes

Previous studies have demonstrated that OPM is a primary pathway to increase AP in soil (Bünemann, 2015; Pistocchi et al., 2018). Based on the metagenomic analysis of microbial genes involved in carbon cycling, the total abundance of OPM-related genes was significantly higher in the BC12 group compared to the BC4 group, which can be attributed to the substantial increase in ratios of C:N and C:P in the BC12 soil. Although biochar supplied P, the large concurrent input of carbon induced a condition of relative P scarcity (Table S1), stimulating an increase in the abundance of OPM-functional microorganisms. This result is consistent with previous studies indicating a significant increase in the OPM by the abundance of bacterial communities under high C:P conditions (Richardson and Simpson, 2011; Yang et al., 2025).

The putative keystone genes involved in OPM were identified using random forest modeling (Figure 3A). Compared to CK, the BC12 group demonstrated a significantly higher total abundance of *phoA* and *phnP* functional genes. According to previous studies, the *phoA* and *phnP* genes play a crucial role in OPM, and the microorganisms containing these genes mediate AP pooling in soil through regulation of phosphatase activity (Wang et al., 2019). Specifically, *phoA* and *phnP* induce the mineralization of various organic P components through hydrolysis of phosphate monoesters and cleavage of C-P bonds, respectively, by secreting P-activating enzymes, therefore affecting the soil’s P availability (Liu et al., 2016; Ma et al., 2020). Furthermore, the P-activation capacity of microbes was significantly increased by the enhanced abundance of *phoA* and *phnP* under low P or P-stressed conditions, thus suggesting that the high dosages of biochar can significantly enhance microbial P activation in P-deficient environments (Zhou et al., 2021).

IPS microorganisms play an essential role in enhancing P availability; however, this study did not show a significant increase in their associated functional genes after biochar amendment (Figure 2), which is likely due to the elevated soil pH. The forms and solubility of soil inorganic P are highly dependent on soil pH (Barrow, 2017). Soil pH gradually increased with increasing biochar, reaching its maximum at 8.66 in the BC12 treatment. Under such alkaline soil conditions, P frequently combines with Ca^2+^ to form insoluble calcium phosphate salts, resulting in a change in IPS (Devau et al., 2011). P-solubilizing microorganisms are required to dissolve these insoluble inorganic forms, which results in investing more resources. In contrast, alkaline phosphatase, a key enzyme in the OPM pathway, has an optimal activity pH within the alkaline range. Therefore, microbial communities likely redirect their metabolic resources toward the OPM pathway, which is more suitable for alkaline environments and can simultaneously obtain P. Furthermore, recent studies indicate that biochar predominantly improves soil AP in alkaline soils by directly increasing soluble P content and indirectly regulating OPM, while the IPS pathway plays a limited role in high-pH soil environments (Chang et al., 2025).

PSD is a fundamental pathway that regulates P pool turnover in the soil (Bai et al., 2020). This study considerably enhanced the abundance of PSD genes in the BC12 group (Figure 2), confirming that the BC12 treatment regulated the storage and release of P by improving PSD functions. Changes in the abundance of keystone functional genes *ppnK* and *relA* contributed to the functional differences in polyphosphate metabolism after biochar treatment. BC12 treatment significantly enriched the *ppnK* and *relA* genes (Table 1). According to previous studies, these genes may significantly regulate the storage and release of soil P by modulating the polyphosphate metabolic networks of soil microorganisms, increasing the AP content. Hildenbrand et al. (2023) reported the catalysis of polyphosphate kinase (encoded by *ppnK*) to transfer a phosphate group between ATP and polyphosphate, facilitating the rapid accumulation of polyphosphate as energy and P pool. Moreover, the *relA* gene, as a core regulator of the stringent response, may enhance the dynamic balance of PSD by controlling intracellular signaling pathways of microbes (Tamman et al., 2020).

Compared to CK, the BC12 group exhibited a significant increase in the relative abundance of *pstA*, *phnD*, and *pstS* genes, likely due to the higher organic C content in BC12 soil (Table 1). According to previous studies, the functional potential of TRA gene clusters was significantly increased in low P stress, which is consistent with the increasing SOC content (Richardson and Simpson, 2011; Dai et al., 2020). Furthermore, Han et al. (2021) reported that the relative abundance of the *pstS* gene was positively correlated with the soil pH. Thus, the BC12 group indicated an abundance of PSD regulatory genes, likely resulting from the synergistic effects of altered SOC content and pH of soil.

### 4.3 Microbial mechanisms driving biochar-mediated P cycling in greenspace soils

According to random forest and microbial co-occurrence networks (Figures 3, 4), four microbial taxa with putative keystone genes for P cycling were identified, which ultimately makes it easier to investigate the microbial mechanisms underlying biochar-mediated regulation of P cycling in urban greenspace soils. These taxa reflected biochar-induced micro-environmental heterogeneity and phylogenetic adaptations, playing a critical role in the transformation of soil P forms, and optimization of soil P supply, maintaining the stability of P cycling functions (Ye et al., 2025). Biochar treatment had a substantial impact on the cumulative relative abundance of two microbial taxa (Modules 1 and 2) in soil. These Modules demonstrated a significant correlation with soil TP, C:P, and NO_3_^−^-N (Figure 5A), according to correlation analysis. Therefore, based on their highest cumulative relative abundance and significant correlation with soil properties, the taxa related to Modules 1 and 2 were defined as the putative keystone microbial taxa. PLS-PM analysis demonstrated that putative keystone microbial taxa were primarily responsible for changes in soil AP (Figures 5B,C). The cumulative relative abundances of Modules 1 and 2 were positively correlated with soil TP (Figure 5A). Putative keystone microbial taxa perform significant functions in soil P cycling (Wang et al., 2023). This investigation illustrated a dual mechanism through which biochar enhances soil AP. Initially, the P present in biochar directly enhances the soil’s P supply, significantly increasing TP and AP (Supplementary Table S1). This enhancement in TP implies an increase in the total pool of transformable P in the soil. Soil expanded P substrate pool sources offer a sufficient foundation of materials for the growth, reproduction, and functional expression of putative keystone microbial taxa involved in soil P cycling (Xu et al., 2019), thus promoting the phosphate-solubilizing microbial enrichment in the soil. Therefore, these enriched phosphate-solubilizing microorganisms likely increased the organic P mineralization, accelerated the conversion of fixed P in the TP pool into soluble, plant-available forms, increasing soil AP levels (Zhang et al., 2025). The results of this study were strongly supported by the positive correlation of TP and putative keystone microbial taxa, along with the significant influence of the microbial taxa on AP (Figure 5). Furthermore, this study revealed the absolute dominant position of bacteria in the studied Modules 1 and 2, comprising both bacteria and archaea. The dominant bacterial genera include: *Lysobacter*, *Polaromonas*, *Rokubacteria*, and *Sphingomonas*. These microbes have been reported to enhance the dissolution and activation of P in the soil, playing a significant role in the soil P cycle (Jin et al., 2025; Lv et al., 2022), simultaneously confirming our conclusion.

### 4.4 Limitations

This study is based on pot experiments using a single plant species. Various plant types may exhibit characteristic microbial interaction patterns, growth habits, and nutrient acquisition strategies, which may affect the response of their related microbial communities to biochar amendment. Moreover, metagenomics methods are limited to exposing the functional potential of microorganisms, while actual gene expression is yet unknown (Terrón-Camero et al., 2022). Future research will focus on the analysis of comparative experiments with multiple plant species, incorporation of multiple sampling time points, and application of techniques such as metatranscriptomics to further elucidate the mechanisms of soil P cycling.

## 5 Conclusion

Biochar treatment, especially BC12, increased the content of soil P and its potential supply in urban greenspace soil. Furthermore, it demonstrated a significant increase in the total abundance of OPM, PSD, REG, and TRA processes-related functional genes, resulting in reshaping the structure of microbial communities associated with these functional genes. Biochar considerably increased AP in urban greenspace soils via two mechanisms: it directly increased soil AP, and elevated soil TP regulates putative keystone P-cycling microbial taxa (Modules 1 and 2), therefore contributing to an even greater increase in soil AP. As the biochar considerably increased the urban greenspace soil AP through clarifying the microbial mechanism, this study advances the understanding of P cycling in greenspace soils.

## Data Availability

The original contributions presented in the study are publicly available. This data can be found here: https://data.mendeley.com/datasets/9gjzgvpz3y/1.
