# Supplementary material for: Biochar regulates putative keystone microbial taxa to drive phosphorus cycling and increase availability in urban greenspace soils
Source: Front Microbiol. 2026 Mar 13;17:1786258. doi: 10.3389/fmicb.2026.1786258 (PMC13021621; doi:10.3389/fmicb.2026.1786258)
Supplement: Supplementary file 1 [file Supplementary_file_1.docx]

**Biochar Regulates Keystone Microbial Taxa to Drive Phosphorus Cycling and Increase Availability in Urban Greenspace Soils**

Kai Pan^1^, Zhenying Zhang^1^, Lingwei Feng^1^, Xiaogang Wu^1^, Xiuyun Yang^1^, Xinping He^2^, Yiqian Xiao^2^, Danning Yang^3^, Chengjiao Duan^3,4^, Qiang Wang^1*^

^1^ College of Forestry, Shanxi Agricultural University, Taigu, Shanxi 030801, China

^2^ China Highway Engineering Consultants Corporation, Beijing, 100089, China

^3^ College of Resources and Environment, Shanxi Agricultural University, Taigu, Shanxi, 030801, China

^4^ State Key Laboratory of Efficient Utilization of Arid and Semi-Arid Arable Land in Northern China, Institute of Agricultural Resources and Regional Planning, Chinese Academy of Agricultural Sciences, Beijing 100081, China

*** Corresponding authors:**

Qiang Wang

E-mail address: qwang@sxau.edu.cn

Postal address: College of Forestry, Shanxi Agricultural University, No. 1 Ming Xian South Road, Taigu 030801, Shanxi, China.

| Treatment | CK | BC4 | BC8 | BC12 |
| --- | --- | --- | --- | --- |
| pH | 8.43±0.09b | 8.43±0.02b | 8.46±0.09b | 8.66±0.15a |
| SOC (g kg^−1^) | 3.43±0.02c | 7.37±2.35b | 8.73±1.81b | 11.62±1.08a |
| TN (g kg^−1^) | 0.31±0.02c | 0.51±0.02b | 0.50±0.05b | 0.58±0.02a |
| TP (g kg^−1^) | 0.48±0.02c | 0.57±0.02b | 0.57±0.03b | 0.62±0.05a |
| C:N | 10.03±0.09b | 15.05±0.95b | 18.04±1.67a | 19.98±1.32a |
| C:P | 6.62±0.89c | 13.96±0.52b | 15.78±1.91b | 18.62±2.01a |
| N:P | 1.39±0.13a | 1.35±0.06a | 1.36±0.13a | 1.26±0.16a |
| DOC (mg kg^−1^) | 79.02±1.96b | 84.49±1.30b | 66.40±1.30c | 76.62±1.60b |
| NO_3_^⁻^-N (mg kg^−1^) | 3.07±0.43c | 5.32±0.97b | 6.93±0.37b | 7.40±1.20a |
| NH_4_^+^-N (mg kg^−1^) | 4.62±0.26ab | 4.38±1.00b | 4.79±0.62ab | 5.56±0.66a |
| AP (mg kg^−1^) | 11.31±0.84c | 14.83±0.92b | 15.30±1.42b | 21.79±1.52a |

**Table S1 Soil basic characteristics of each treatment**

Note: CK, control; BC4, 4% biochar; BC8, 8% biochar; BC12, 12% biochar. SOC, soil organic carbon; TN, total nitrogen; TP, total phosphorus; C:N, SOC/TN; C:P, SOC/TP; N:P, TN/TP; DOC, dissolved carbon; NO_3_^⁻^-N, nitrate nitrogen; NH_4_^+^-N, ammonium nitrogen; AP, available phosphorus.

**Table S2 Taxonomic classification of microbes in Modules 1 and 2**

| Module 1 | | Module 2 | |
| --- | --- | --- | --- |
| Bacteril | Archaeal | Bacteril | Archaeal |
| *Lysobacter* | *Nitrosopumilus* | *Rokubacteria* | *Nitrososphaeraceae* |
| *Polaromonas* | *Methanoperedenaceae* | *Sphingomonas* | *Nitrososphaera* |
| *Hyphomicrobium* |  | *Nitrospira* | *Nitrosocosmicus* |
| *Pseudorhodoplanes* |  | *Candidatus_Gaiellasilicea* | *Archaea* |
| *Steroidobacter* |  | *Anaerolinea* |  |
| *Gemmatirosa* |  | *Parcubacteria* |  |
| *Sulfuritalea* |  | *Conexibacter* |  |
| *Pyxidicoccus* |  | *Streptomyces* |  |
| *Ramlibacter* |  | *Gaiella* |  |
| *Usitatibacter* |  | *Gemmatimonas* |  |
| *Tectomicrobia* |  | *Binatia* |  |
| *Nitrosospira* |  | *Candidatus_Omnitrophota* |  |
| *Pseudonocardia* |  | *Solirubrobacter* |  |
| *Pirellula* |  | *Rubrivivax* |  |
| *Methylovorus* |  | *Roseisolibacter* |  |
| *Pedosphaera* |  | *Pyrinomonas* |  |
| *Syntrophus* |  | *Latescibacteria* |  |
| *Microvirga* |  | *Methyloceanibacter* |  |
| *Nitricoxidivorans* |  | *Thermoleophilum* |  |
| *Cupriavidus* |  | *Gloeobacter* |  |
| *Exilibacterium* |  | *Rubrobacter* |  |
| *Reyranella* |  | *Caldimonas* |  |
| *Phytoactinopolyspora* |  | *Propionivibrio* |  |
| *Rhabdaerophilum* |  | *Baekduia* |  |
| *Candidatus_Denitrolinea* |  | *Frankia* |  |
| *Methyloversatilis* |  | *Rhodoplanes* |  |
| *Cyanobacteriota* |  | *Candidatus_Microthrix* |  |
| *Aridibacter*  *Bauldia*  *Herpetosiphon*  *Magnetospirillum*  *Ardenticatena*  *Knoellia*  *Nitrosopumilus*  *Candidatus_Nealsonbacteria*  *Phenylobacterium*  *Microbulbifer* |  | *Methylobacillus*  *Aurantimonas*  *Novosphingobium*  *Nonomuraea*  *Sulfuricella*  *Methylomonas*  *Stella*  *Nitriliruptor*  *Devosia*  *Arenimonas* |  |

Note: Arrange from high to low according to the magnitude of relative abundance.
**Continued table S2**

| Module 1 | | Module 2 | |
| --- | --- | --- | --- |
| Bacteril | Archaeal | Bacteril | Archaeal |
| *Candidatus_Hydrogenedentes*  *Parvibaculum*  *Agromyces*  *Nitrospina*  *Methylibium*  *Aquabacterium*  *unclassified_o__Pseudanabaenales*  *Aromatoleum*  *Thioalkalivibrio*  *Afipia*  *Desulfuromonas*  *Anatilimnocola*  *Candidatus_Andersenbacteria*  *Candidatus_Melainabacteria*  *Methylocaldum*  *Stutzerimonas*  *Sinorhizobium*  *Anaeromyxobacter*  *Methylophilus*  *Capillimicrobium*  *Paraconexibacter*  *Spongiibacter*  *Gracilinema*  *Acidiferrimicrobium*  *Lamprocystis*  *Flavobacterium*  *Labilithrix*  *Ancylobacter*  *Ideonella*  *Litorilinea*  *Candidatus_Dormibacter*  *Thermomicrobium*  *Pontibacter*  *Longimicrobium*  *Chryseolinea*  *Comamonas*  *Stagnimonas*  *Candidatus_Sumerlaeaceae*  *Azohydromonas*  *Minwuia*  *Aeromicrobium* |  | *Geminicoccus*  *Thermalbibacter*  *Gimesia*  *Sphaerobacter*  *Salinibacterium*  *Solimonas*  *Nostoc*  *Aestuariivirga*  *Caldimicrobium*  *Calothrix*  *Mycoplana*  *unclassified_c__Gammaproteobacteria*  *Henriciella*  *Polynucleobacter*  *Vineibacter*  *Fimbriimonas* |  |

**
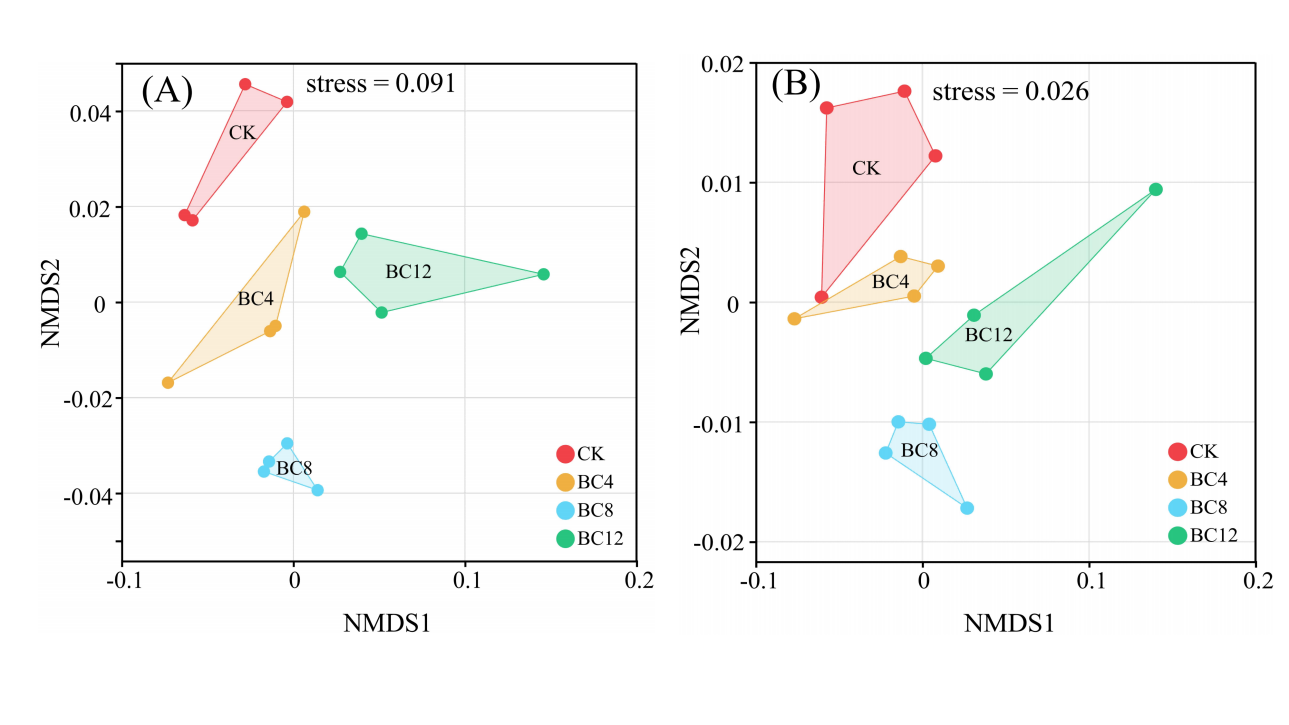
**

**Fig. S1.** Non-metric multidimensional scaling (NMDS) analysis of soil P cycling functional microbial. (A) P-cycling functional microbial at the genus level; (B) P-cycling functional microbial at the gene level.

Note: CK, control; BC4, 4% biochar; BC8, 8% biochar; BC12, 12% biochar.
